# Psychotherapy Training: Educationally Beneficial or Simply Anxiety Inducing?

**DOI:** 10.1192/bjo.2025.10261

**Published:** 2025-06-20

**Authors:** Jelena Buracionok, Ye Do, Isabel Mark

**Affiliations:** South-West London and St George’s Mental Health NHS Trust, London, United Kingdom

## Abstract

**Aims:** Developing core psychotherapy knowledge and skills is considered a key part of the Royal College of Psychiatrists curriculum. This cross-sectional study aimed to evaluate the current provision of psychotherapy teaching and supervision for core trainees within South-West London and St George’s Trust, identifying key concerns and proposing improvements to enhance the training experience.

**Methods:** 27 trainees participated in a trust-wide questionnaire, achieving a 58% response rate amongst core trainees in years two and three. The questionnaire was conducted online using Microsoft Forms, with a link distributed via email, including multiple-choice and open-ended questions, and focusing on the feelings trainees have towards psychotherapy teaching as well as the benefits and challenges they have observed. Data were analysed using Microsoft Excel and a thematic approach.

**Results:** A general feeling of anxiety was described by many, with some trainees describing the current system as “frustrating and disheartening”. Other key themes were the perceived lack of preparedness before starting psychotherapy cases, variation in expectations of supervisors and case allocation delays. 87.5% of trainees felt they lacked sufficient knowledge in psychotherapy before starting short-cases, 72% for long-cases. 56% stated they were providing or planning to provide sessions out of their working hours. Reasons given included fears of upsetting the patient, not providing continuity of care during their annual leave or zero days and not being able to complete their case in time for training requirements. 11% were dissatisfied with their short-case experience, 50% with their long-case experience. 61% of participants felt they would benefit from private therapy themselves, to support them during the process.

**Conclusion:** Results highlight concerns that trainees can have in psychotherapy training, which are being addressed locally but can also have relevance for other training programmes and practices. The perceived lack of adequate teaching prior to starting cases is striking, and could pose potential risks to patient safety. The high numbers of trainees who report offering therapy outside working hours poses risks to trainee well-being, as well as insurance coverage and patient safety. This study also revealed how a large proportion of trainees believe they would benefit from being offered personal psychotherapy, something that is not routinely offered in training. Enhancing psychotherapy training with timely, comprehensive and structured support, whilst also considering trainee wellbeing, could lead to improved educational impact, benefiting both trainee professional development and ultimately patient care.

